# Implementing long-acting injectable HIV pre-exposure prophylaxis services at private pharmacies in Kenya: client, pharmacy provider and key stakeholder perspectives on potential challenges and opportunities

**DOI:** 10.1136/bmjgh-2025-019210

**Published:** 2026-01-28

**Authors:** Stephanie D Roche, Kevin Kamolloh, Nicholas Thuo, Maurice Opiyo, Vallery Ogello, Alfred Odira, Emmah Owidi, Perez Ochwal, Marion Hewa, Lydia Adiema, Felix Mogaka, Victor O Omollo, Rachel C Malen, Kendall Harkey, Jenell Stewart, Kenneth Ngure, Katrina F Ortblad, Elizabeth A Bukusi

**Affiliations:** 1 Public Health Sciences Division, Fred Hutchinson Cancer Center, Seattle, Washington, USA; 2 Centre for Microbiology Research, Kenya Medical Research Institute, Kisumu, Kenya; 3 Partners in Health and Research Development, Thika, Kenya; 4 Hennepin Healthcare, Minneapolis, Minnesota, USA; 5 Department of Global Health, University of Washington, Seattle, Washington, USA; 6 School of Public Health, Jomo Kenyatta University of Agriculture and Technology, Nairobi, Kenya

**Keywords:** HIV, Kenya, prevention strategies, qualitative study, delivery of health care

## Abstract

**Introduction:**

Maximising the impact of new and forthcoming long-acting injectable HIV pre-exposure prophylaxis (PrEP) products will require novel delivery approaches that widen accessibility and prioritise clients’ needs and preferences. To understand the potential barriers and facilitators to delivering injectable PrEP via private pharmacies in Kenya, we conducted qualitative formative research.

**Methods:**

From July to September 2023, we interviewed pharmacy providers, pharmacy clients and other key stakeholders of HIV service delivery in Central and Western Kenya. Our purposive sample included some providers and clients with prior experience delivering or obtaining oral PrEP at a pharmacy as part of a pilot study and some without such experience. We analysed verbatim transcripts thematically using a combination of inductive and deductive approaches, the latter informed by the Consolidated Framework for Implementation Research.

**Results:**

We interviewed 25 pharmacy clients, 16 pharmacy providers and nine key stakeholders. Each group was ~50% female, and median age among clients was 25 (IQR 23–29). Overall, participants supported the idea of pharmacy-based injectable PrEP delivery. Anticipated facilitators included perceived benefits of injectable over oral PrEP; characteristics of pharmacies (eg, long operating hours) that could fulfil clients’ need for accessible, fast and private injectable PrEP services; and existing skillsets of pharmacy providers, especially those already trained on injectable contraception. Anticipated barriers included gaps in enabling policy; pharmacies’ lack of integration with the public health sector, such as its health information system; low client knowledge of and/or ability to pay for injectable PrEP and pharmacy staffing and compensation structures that could disincentivise providers.

**Conclusions:**

Participants in this study expressed cautious optimism that private pharmacies could be an effective delivery channel for injectable PrEP in Kenya. If private pharmacies facilitate access to and use of injectable PrEP, they could play a pivotal role in ending HIV as a public health threat.

**WHAT IS ALREADY KNOWN ON THIS TOPIC**

- Recent studies in Kenya on oral pre-exposure prophylaxis (PrEP) delivery via private pharmacies suggest that this delivery channel reaches many individuals who could benefit from HIV prevention services and are willing to pay for the convenience and privacy.
- No study has explored the potential of private pharmacies to deliver long-acting injectable PrEP (LAI PrEP) products, which modelling studies suggest could substantially reduce global HIV incidence if rolled out at scale through differentiated service delivery models.

**WHAT THIS STUDY ADDS**

- This study is the first to systematically identify potential barriers and facilitators to private pharmacy-delivered LAI PrEP services, as anticipated by Kenyan pharmacy clients, pharmacy providers and other key stakeholders.

**HOW THIS STUDY MIGHT AFFECT RESEARCH, PRACTICE OR POLICY**

- The findings provide timely evidence to inform Kenya’s national implementation plan for LAI PrEP introduction (eg, policy gaps that would need to be addressed to enable pharmacy delivery) and identify future research directions for this novel delivery channel.

## Introduction

 After a decade of suboptimal access and uptake of oral HIV pre-exposure prophylaxis (PrEP) worldwide,^1^ the advent of highly effective long-acting injectable (LAI) PrEP products has brought renewed optimism to the HIV prevention landscape and a keen interest in novel delivery approaches that might accommodate multiple PrEP products while supporting clients’ informed choice.^2 3^ To date, 53 countries—including 13 in sub-Saharan Africa (SSA)—have approved bimonthly intramuscular injections of long-acting cabotegravir (CAB-LA) for HIV prevention,^4^ and in June 2025, the USA approved twice-yearly subcutaneous injections of lenacapavir (LEN),^5^ which was found highly efficacious and safe in the PURPOSE 1 and 2 trials.^6 7^ These products may be especially impactful in SSA, where half of the world’s new HIV infections (~660 000/1.3 million) occurred in 2022.^8^

To accelerate access to LAI PrEP, HIV prevention advocates have called for its roll-out to incorporate learnings from that of oral PrEP.^4^ For example, to accommodate a known preference among many clients to access PrEP in non-clinical settings,^9^ countries will need to adapt where LAI PrEP services are delivered and by whom, in line with principles of differentiated service delivery.^10 11^ Another key lesson learnt from oral PrEP is that limiting initial roll-out to key populations (eg, sex workers) can create PrEP stigma.^12 13^ To avoid this, some have called for countries with generalised HIV epidemics to ‘normalise’ LAI PrEP^14^ by availing it from the outset to members of the general population who could benefit—as Zambia^15^ is doing—and delivering it in places where the general population accesses health services.

Due to global shortages of CAB-LA,^15^ its programmatic roll-out is still nascent. Currently, most countries with CAB-LA supply are prioritising traditional clinical settings for initial roll-out, with injections administered by nurses or doctors.^16^,^18^ However, early examples of differentiated CAB-LA delivery in the USA include injection administration task-shifted to community health workers at a single primary care centre in Washington, District of Columbia^19^ and to pharmacists at a single pharmacy in Seattle, Washington.^20^

Faced with declining donor funding for its national HIV programme,^21 22^ Kenya is exploring ways to leverage the private sector for HIV service delivery,^23^ including the largely untapped well of human and physical resources comprising its private pharmacy sector.^23 24^ This sector boasts approximately 11 000 licensed pharmaceutical technologists; 2600 licensed pharmacists and 7400 registered pharmacies,^25^ the vast majority of which are for-profit and financed by clients’ out-of-pocket payments.^26 27^ Comprehensive information on Kenya’s private pharmacy sector—including its clientele—is lacking; however, findings from Kenya’s Demographic and Health Survey (DHS) and several Kenya-based research studies suggest that private pharmacies are routinely accessed for sexual and reproductive health products. For example, Kenya’s 2022 DHS found that, among women aged 15–49 who used male condoms (n=1227), the contraceptive pill (n=1710) or emergency contraception (n=206) in the past 12 months, a sizeable portion of respondents—42%, 52% and 87%, respectively—purchased the method at a private pharmacy.^28^ Other studies have found that private pharmacies are a common source for at-home pregnancy tests,^29 30^ HIV self-tests^31^ and sexual performance-enhancing drugs.^32^ Lastly, recent pilot studies of pharmacy-delivered oral PrEP services in Kenya have demonstrated that private pharmacies reach diverse populations that engage in behaviours associated with HIV risk acquisition (eg, young single men; middle-aged married women) and observed high uptake and acceptability of PrEP among pharmacy clients and providers.^33^,^36^

Kenya approved CAB-LA in June 2024^37^ and, as one of nine countries in SSA being prioritised for LEN introduction by the Global Fund,^38^ it is anticipated to approve LEN in 2026.^39^ Meanwhile, the Kenya MOH is developing an implementation plan for LEN introduction and subsequent scale-up, with an eye towards community-based delivery models and a total market approach that leverages the comparative strengths of the public, private for-profit and private non-profit sectors to meet population needs. To generate timely evidence to inform these guidelines and the design of a pilot study, we conducted formative qualitative research with Kenyan pharmacy clients, pharmacy providers and other key stakeholders. Our objective was to identify potential barriers and facilitators to delivering LAI PrEP via private pharmacies in Kenya.

## Methods

### Setting and participants

This study took place in Kisumu and Kiambu Counties of Kenya, where HIV prevalence is 15% and 2%, respectively.^40^ We used quota sampling to purposively select PrEP clients, pharmacy providers and other key stakeholders (all aged 18+). Eligible clients self-reported purchasing health products at pharmacies and using oral PrEP within the past 6 months. Eligible pharmacy providers were licensed pharmacists or pharmaceutical technologists (hereafter, ‘pharm techs’). Eligible stakeholders were individuals with direct involvement in HIV-related or pharmacy-related policymaking, regulation and/or programme implementation in Kenya.

To capture perspectives from individuals with prior experience obtaining or delivering oral PrEP services at pharmacies, we drew two-thirds of clients and half of providers from individuals who had participated in a pilot study^34^ 6 months prior; research assistants (RAs) randomly selected providers as well as clients who refilled and never refilled PrEP during the pilot and contacted them via phone. The remaining PrEP clients and pharmacy providers were recruited in person from public health facilities and licensed pharmacies in the study counties. For our client sample, we aimed to have an even distribution of men and women above and below 25 years of age. Stakeholders were recruited through the research team’s professional networks via phone or email. We determined our sample size based on the concept of information power,^41^ which posits that the adequacy of a qualitative sample depends on the information it holds relative to the study’s aims, rather than on reaching a fixed number of participants. Given the tightly scoped nature of our research question, the specificity and relevance of our sample to the research question and anticipated quality of dialogue between interviewees and qualitative researchers, we anticipated that conducting eight to 10 interviews per subcategory (for a total of ~50 interviews) would yield highly relevant, focused information sufficient for achieving our research objective.

### Data collection

We developed semi-structured interview guides (online supplemental appendix A) informed by the updated Consolidated Framework for Implementation Research (CFIR V.2.0)^42^ and findings from previous studies on pharmacy-delivered oral PrEP services in Kenya.^33 34 43 44^ A meta-framework of multiple existing implementation theories, the CFIR V.2.0 features a comprehensive set of multilevel determinants (ie, barriers and facilitators at the individual, organisational and community level) that impact implementation of evidence-based interventions. Embedded within the CFIR V.2.0 is the Communication Opportunity Motivation-Behavior (COM-B) model by Michie *et al*, which posits that, for any behaviour to occur, a person must have the capability (eg, skills) to carry out the behaviour, opportunity (eg, autonomy) to make the behaviour possible and the motivation (eg, commitment) that energises and directs the behaviour.^45^ After presenting participants with a brief, standardised description of CAB-LA and LEN (eg, injection type and site, common side effects), we asked participants about their anticipated willingness and ability to obtain, deliver or support LAI PrEP services at pharmacies and specifically probed about potential barriers and facilitators related to the innovation itself (LAI PrEP); the proposed inner setting (private pharmacies); the outer setting (eg, Kenyan policy environment) and the capability, opportunity and motivation of key individuals (eg, pharmacy clients and providers) to engage with the intervention as intended. We also solicited possible solutions to participant-identified barriers and recommendations. Experienced Kenyan qualitative RAs (authors NT, MO, VO, AO, PO, MH and LA) conducted interviews in participants’ preferred language (English, Kiswahili or Dholuo) in a private location of the participant’s choosing or over the phone. Interviews were audio recorded and transcribed verbatim. After each interview, RAs wrote debrief reports summarising key points.

### Analysis

We analysed data thematically using a combination of inductive (ie, data-driven) and deductive (ie, framework-driven) approaches, informed by principles of rapid qualitative analysis.^46 47^ After repeated readings of debrief reports to identify emergent barriers and facilitators, author SDR drafted a summary template (online supplemental appendix B) for capturing key findings, structured around the following topics informed by the CFIR V.2.0: policy, regulation and monitoring; available resources; provider competency, training and support; factors affecting client willingness and ability to use pharmacy-delivered LAI PrEP services (eg, awareness of LAI PrEP; access to pharmacies; trust in pharmacies/pharmacy providers; anticipated quality of care). The summary template also included space for illustrative quotes and the analyst’s questions and observations. To establish a shared understanding of the summary template and consistency in its application across analysts, the team reviewed several example summaries together and refined the template based on consensus. After multiple readings of the interview transcripts, seven team members (authors NT, MO, VO, AO, PO, MH and LA) independently completed one summary per interview in an electronic data collection platform (Research Electronic Data Capture), with each summary checked and updated, as needed, by a second team member. Throughout the analysis, the team met routinely to resolve discrepancies in template application. Final summaries were exported in matrix format. Three team members (authors SDR, KH and RCM) read across and within summaries independently to identify additional patterns; consulted original transcripts for context, as needed and collapsed or expanded key categories through consensus. Online supplemental appendix C contains additional details about our methodology, as per the Consolidated Criteria for Reporting Qualitative Studies checklist.

### Patient and public involvement and ethics

Neither patients nor the public were directly involved in the design or conduct of this study; however, the focus of this research—understanding the potential barriers and facilitators to implementing LAI PrEP delivery at private pharmacies—is responsive to broader calls from patients and HIV advocacy groups to make PrEP services available outside of traditional clinic settings. All participants were compensated 500 Kenyan shillings (~US$5) per interview.

## Results

### Participants

From July to September 2023, we interviewed 24 PrEP clients, 16 pharmacy providers and nine key stakeholders (table 1). Median interview duration was 1 hour and 2 min (IQR 50–70 min). About half of clients were female and under the age of 25 years. Among clients who never refilled PrEP at the pharmacy, all attributed their discontinuation to personal circumstances (eg, change in perceived HIV risk) and not to the delivery venue. About two-thirds of pharmacy providers were female, and their median age was 35 years. Among stakeholders, two-thirds were female, the median age was 44 years and about half worked for government and half for non-governmental organisations.

**Table 1:** Demographics of participants who completed in-depth interviews

Characteristic	PrEP clients (n=24)	Pharmacy providers (n=16)	Other key stakeholders (n=9)
Female*	13 (54%)	10 (63%)	6 (67%)
Age†, median (IQR)	25 (23–29)	35 (33–38)	44 (38–51)
Unmarried	17 (71%)		
Education: secondary or higher	20 (83%)	16 (100%)	
Prior oral PrEP experience*			
Clinic-delivered services only	8 (33%)		
Pharmacy-delivered services, never refilled‡§	8 (33%)		
Pharmacy-delivered services, refilled 1+ time‡	8 (33%)		
Cadre			
Pharmaceutical technologist		11 (69%)	
Pharmacist		5 (31%)	
Prior experience delivering oral PrEP*‡		8 (50%)	
Years in pharmacy work, median (IQR)		11 (7–13)	
Current or prior experience administering injections		13 (81%)	
Their pharmacy sells and/or administers the following injection(s):			
Injectable contraception		12 (75%)	
Tetanus vaccine		7 (44%)	
Rabies vaccine		7 (44%)	
Other¶		5 (31%)	
Employer			
Kenya Ministry of Health			3 (33%)
Pharmacy professional body			3 (33%)
Pharmacy regulator			2 (22%)
PrEP implementing partner			1 (11%)

* Characteristic used during purposive sampling to achieve the distribution shown.

† Client interviewees purposively sampled so half were under 25 years of age.

‡ As part of the Pharm PrEP Pilot Extension Study, which ran from January to July 2022 at 12 pharmacies in Kisumu and Kiambu Counties, Kenya.

§ Among these eight clients, self-reported reasons for not refilling oral PrEP at the pharmacy included: reduced HIV risk so felt PrEP no longer needed (n=2); moved to new county and travel distance too far (n=2); inadvertently missed follow-up appointment (n=2; of note, these clients only managed to return to the pharmacy after the study had ended and were referred to nearby public health facilities, where both re-initiated PrEP services); pill burden (n=1) and PrEP side effects (n=1).

¶ Other injections offered at these five providers’ pharmacies include anti-D immunoglobulin injection, insulin injection, injectable painkillers (eg, tramadol), injectable antibiotics (eg, metronidazole) and vaccines for influenza and hepatitis.

PrEP, pre-exposure prophylaxis.

We present the potential barriers and facilitators participants identified, with those pertaining to the CFIR domains of innovation, outer setting and inner setting summarised in table 2 and those pertaining to the individuals domain summarised in table 3.

**Table 2:** Potential barriers and facilitators to pharmacy-delivered injectable PrEP in the innovation, outer setting and inner setting domains of the Consolidated Framework for Implementation Research

Construct	Anticipated barrier (–) or facilitator (+) (*quote ID*)	Illustrative quotes
Domain: innovation		
Relative advantage	(+) Anticipated advantages of injectable over oral PrEP: Elimination of pill burden supports adherence and gives greater peace of mind about HIV (*A*) Fewer annual visits; decreases client and provider burden (*B*) Easier to use without others knowing (*C*) (–) Anticipated disadvantage: discomfort with injections, especially in the abdominal area for fear of harm to fertility (*D*)	“You can forget to swallow the [PrEP] tablets, but at least if you get a [CAB-LA] injection … you know that you are safe for 2 months”. (*Male clinic PrEP client, age 21 years, ID #19*) “[Even] if they [lenacapavir clients] consume up to one hour [of your time], you won’t see them again for six months, so for the rest of the months, they will not consume your time. So that will be very good”. (*Female pharm tech with PrEP delivery experience, age 38 years, ID #34*) “In the previous study, there were those [clients] who would pass by [the pharmacy] every morning to take their [PrEP] pill because they did not want anyone to find them taking a drug every day. So this [injection] will be more confidential”. (*Female pharm tech with PrEP delivery experience, age 35 years, ID #33*) “Can it lead to impotence? That is what I would ask myself [about lenacapavir]. … [The injection site] is so close to the reproductive system”. (*Male pharmacy PrEP client who refilled, age 29 years, ID #24*)
Domain: outer setting		
Policy and laws	(–) Current gaps in Kenyan policy: Lack of national injectable PrEP guidelines Pharmacies not an approved level of healthcare system for ARV delivery Pharmacy provider scope of practice does not include injections (*A*) (+) Precedent: policy approach used to enable pharmacy delivery of injectable contraception could serve as blueprint for injectable PrEP (*B*)	“The role of pharmacists and pharmaceutical technologists is outlined in the *Health Act*. … So in terms of administering the injection, this [expansion of scope to include injection administration] would need to be specified”. (*Male pharmacist with no PrEP delivery experience, age 29 years, ID #37*) “If it [injectable PrEP] becomes a cluster two [Schedule II drug], it can be given by a pharmacy practitioner. Since we have done this with the family planning one [Depo-Provera injection], it will be easier [to do for injectable PrEP]. … We have already trained them to give the Depo [injection]”. (*Male stakeholder from Kenya’s pharmacy regulatory agency, age 63 years, ID #49*)
Domain: inner setting		
Physical infrastructure	(–) Some pharmacies lack private rooms	“Having a private room would be a limiting factor for many pharmacies. … At my pharmacy right now, there is no private room”. (*Female pharmacist with no PrEP delivery experience, age 29 years, ID #39*)
Work infrastructure	(–) Typical staffing levels—one pharmacy provider per shift—not conducive to continuous delivery of high-quality injectable PrEP services	“During the evening, it [delivering oral PrEP] was more tricky because I was alone. So I would take the [PrEP] client very fast in this private room because … I still had customers. [To deliver injectable PrEP] two [staff] per shift [would be ideal]”. (*Female pharm tech with PrEP delivery experience, age 35 years, ID #33*)
Access to knowledge and information	(–) Pharmacies lack connection to public health system for consultations, referrals and technical assistance (+) Precedent: in pilot studies, pharmacy providers liked having access to a remote clinician and used this resource appropriately (+) Kenya MOH is open to providing supportive supervision to pharmacies (*A*)	“Once it is allowed that they [pharmacies] do it [deliver injectable PrEP], then we [the MOH] will ensure that such pharmacies are incorporated … into our routine supervision and mentorship sessions”. (*Female stakeholder from Kenya MOH, age 55 years, ID #43*)
Compatibility	Procurement: (–) How pharmacies would source drug is still a grey area; possible issues: cost and time burden (*A*) Documentation and reporting: (–) Pharmacies do not typically maintain detailed client records or do reports (*B*) (–) Current PrEP reporting tools are long (+) Pharmacovigilance reporting system already exists Sharps disposal: (+) Sharps disposal via public clinics already exists (–) Existing system not always functional (*C*) (–) Existing system requires pharmacies to drop off waste Client follow-up: (+) Many pharmacies already call/text clients due for refill (–) This approach is prone to failure (eg, clients’ contact info changes) (*D*)	“It was time-consuming [going to the health facility to pick up the PrEP commodities]. … [For injectable PrEP] I—the person who is giving the injection—should not be the same one going to pick [the drugs]. … A delivery person is required”. (*Female pharm tech with PrEP delivery experience, age 38 years, ID #34*) “Filling this book [about PrEP clients served] was tedious work, and it also needed a lot of effort to understand each and every book [field] and what to write on it. So, the recording work was too much”. (*Female pharm tech with PrEP delivery experience, age 40 years ID #35*) [Currently] we rely so much on the hospitals nearby [for sharps disposal], and they are not reliable. … Sometimes you may have to stay with the waste for months. … It is a national issue that the county governments should look at. (*Male pharm tech with PrEP delivery experience, age 61 years, ID #26*) “Someone might forget the date to come back, so you’ll have to remind them … But it will be hard. You know, there are those who have numbers that can destroy [stop working] any time”. (*Female pharm tech with PrEP delivery experience, age 27 years, ID #28*)
Available resources	(+/–) Pharmacies could easily procure most supplies, but willingness to invest in these will depend on demand and profitability (*A*) (+/–) Pharmacies lack injectable PrEP drug but are open to getting it directly from Kenya MOH, ideally for free or at a subsidised cost (*B*)	“Couches are not that cheap. We have to be sure of the inflow of the [injectable PrEP] patients and whether it [demand for injectable PrEP] is long-term to [justify] invest[ing] in a couch”. (*Male pharm tech with PrEP delivery experience, age 61 years, ID #26*) “If I have the [injectable PrEP drugs] and the HIV [testing] kits being given to me at no cost, there will be no problem [delivering it]”. (*Female pharm tech with PrEP delivery experience, age 34 years, ID #25*)

ARV, antiretroviral; MOH, Ministry of Health; PrEP, pre-exposure prophylaxis.

**Table 3:** Potential barriers and facilitators to pharmacy-delivered injectable PrEP in the individuals domain of the Consolidated Framework for Implementation Research

Construct	Anticipated barrier (−) or facilitator (+) (*quote ID*)	Illustrative quotes
Client need	(+) Pharmacy delivery would fulfil some clients’ need for HIV services that are: Accessible (*A*) Fast/Efficient (*B*) Private/Discreet Respectful/Non-judgemental	“It is easier [getting PrEP at a pharmacy]. Most pharmacies close around 8 or 9 pm … and are open throughout the week, unlike clinics, which are open maybe 5 days a week … and maybe you go to get [PrEP] and you find that they have closed already”. (*Male pharmacy PrEP client who refilled, age 46 years, ID #12*) “[At public clinics] you will have to go through three people before you reach the doctor who is prescribing the medication to you. But if you go to the pharmacy, you go directly to the person who will serve you what you want”. (*Male pharmacy PrEP client who refilled, age 29 years, ID #24*)
Client capability	(−) Low client awareness of injectable PrEP (−) Low client awareness of its availability at pharmacies (*A*)	“People won’t know such [injectable PrEP] is offered at the pharmacies. They only know of the hospitals. … So we need a bit of advertising or maybe a banner”. (*Male pharmacist with prior PrEP delivery experience, age 35 years, ID #38*)
Client opportunity	(+/−) Cost: amount pharmacies charge will influence affordability for clients; the government could subsidise delivery and put price caps (*A*) (+/−) Quantity and geographic distribution of pharmacies offering injectable PrEP will influence clients’ access (*B*)	“You can never tell how much a chemist will ask for. … They might say they will be charging 200 [shillings], and you might fail to get that 200. So it will be a problem for those of us who don’t have the ability to pay”. (*Female pharmacy PrEP client who refilled, age 38 years, ID #23*) “When I came to [the pharmacy to] pick those [oral PrEP] medications, it was when I had activities to do in [this area]. It needed transport because it is a bit far. … These PrEPs should be available in many other pharmacies. … Every region should have these injectables. They should not just be available in one place”. (*Male pharmacy PrEP client who refilled, age 29 years, ID #24*)
Client motivation	(+/−) Trust in injectable PrEP (*A*) (+/−) Confidence in provider’s integrity (*B*) (+/−) Confidence in provider’s competence, especially knowledge, counselling and injection skills (*C*)	“[I’d need to know] if it prevents [HIV] 100% and if it’s safe”. (*Female pharmacy PrEP client who refilled, age 23 years, ID #21*) “[Providers] should not be someone who sees me in town and starts saying, ‘That is my client. I gave that one medicine.’ … I would never go there”. (*Male clinic PrEP client, age 21 years, ID #7*) [The provider] should be able to tell you the benefits of injectable PrEP over oral pills. They must explain to you why they have upgraded from the oral pills to this [injectable] one. (*Female pharmacy PrEP client who did not refill, age 25 years, ID #13*)
Provider capability	(+) *For lenacapavir:* some pharmacy providers already administer subcutaneous injections; a few have been trained by the MOH to give injectable contraception (*A*) (+/−) A small number of pharmacies have nurses/doctors; requiring these cadres would disqualify most pharmacies (*B*) (+) Existing trainings (eg, on injectable contraception delivery) could be repurposed	“Most licensed pharmacies already offer subcutaneous injections, like insulin. So the knowledge is there. What we are lacking is the [lenacapavir] drug”. (*Male pharmacist with no PrEP delivery experience, age 31 years, ID #31*) “You can have a nurse [administer the injection]. … I have two licensed nurses [on staff], but I think not all pharmacies can afford to do what we are doing. (*Male pharm tech with PrEP delivery experience, age 38 years, ID #36*)
Provider opportunity	(−) Amount of time it takes to deliver injectable PrEP may, at times, exceed what pharmacy providers have available	“Because of the high cost of overheads, we want to serve as many people in a day as possible and also to reduce the time of serving patients. … If you’re to go through the whole process of having to test patients for HIV and to counsel them on the injectable and everything else, it means taking your time with one patient, [which] I think would be a challenge”. (*Male pharmacist with no PrEP delivery experience, age 29, ID #37*)
Provider motivation	(−) Low profit margin and high time burden could weaken pharmacy commitment to delivery (*A*) (−/+) A commission-based compensation structure might disincentivise providers from delivering injectable PrEP; an incentive paid directly to the provider delivering the service might counteract this issue (*B*)	“A pharmacy is a business. I can’t spend time with you as a client for, like, 1 hour when I could have made maybe 10 000 or 5000 shillings selling [other] medicine”. (*Female pharm tech with PrEP delivery experience, age 38 years, ID #34*) “We work with these [sales] targets. If you achieve your target, there is a certain amount of money [bonus commission] you are given … So if [after spending time delivering injectable PrEP] there is something you are compensated [directly] at the end of it all yourself, … that will give me more comfort. Even if I don’t get the other one [the bonus commission], I still get something out of this [injectable PrEP delivery]”. (*Female pharm tech with PrEP delivery experience, age 40 years, ID #35*)

MOH, Ministry of Health; PrEP, pre-exposure prophylaxis.

### Innovation domain

#### Relative advantage

Participants from all three groups anticipated three key advantages of LAI PrEP compared with oral PrEP. First, they thought the elimination of daily pill-taking would facilitate adherence and bring clients greater peace of mind about acquiring HIV. Second, providers and stakeholders thought that twice-yearly, compared with quarterly, clinic visits would reduce burden on clients and providers, making it easier for them to access or deliver services. Lastly, clients and providers felt injections would be more discrete than oral pills, making it easier for clients to keep their PrEP use private.

However, several clients and providers anticipated that some clients would continue to prefer oral PrEP over CAB-LA due to discomfort exposing part of their buttocks in a pharmacy setting. For LEN, nearly all clients said they would be hesitant to get an injection in the abdominal area due to fear of adverse effects on fertility:

[The abdomen is] the same place where I carry my pregnancy. If at all I could have been pregnant and still not aware, and they inject that place, I am afraid it may affect me. The one for the buttocks [CAB-LA] is better. (*Female clinic PrEP client, age 26 years, ID #5*)

### Outer setting domain

#### Policy and laws

Providers and stakeholders identified gaps in Kenyan policy that would need to be addressed to enable pharmacy delivery of LAI PrEP. Participants from both groups called for developing LAI PrEP guidelines that specify pharmacies as a target delivery channel and which cadres should deliver it, noting that for pharmacy providers to deliver it would likely require expanding pharmacists’ and/or pharm techs’ scope of practice—outlined in the Good Pharmacy Practice Guidelines—to include injection administration. A few stakeholders suggested using an approach similar to that which enabled pharmacy-delivered injectable contraception in Kenya: rescheduling the drug so it can be initiated by pharmacy providers and creating a certificate training programme. Stakeholders also reported that the current Kenya Essential Medicine List would need to be revised to include LAI PrEP and indicate that these drugs should be available for use (ie, distributed, stored, prescribed and dispensed) at the pharmacy level of the healthcare system. Lastly, providers and stakeholders reported the need to establish minimum criteria that pharmacies must meet to deliver LAI PrEP and recommended these include a current government licence and registration, private consultation room and a provider certified in LAI PrEP delivery.

### Inner setting domain

#### Physical infrastructure

Nearly all participants from all three groups said that pharmacies should be required to have a fully private room to ensure client privacy and comfort. Some providers noted that this requirement might disqualify a considerable number of pharmacies in Kenya.

#### Work infrastructure

Providers reported that most pharmacies have only one pharmacy provider working per shift and identified this staffing level as a potential barrier to making LAI PrEP services available continuously throughout the day.

#### Access to knowledge and information

Nearly all stakeholders and providers—especially those with prior experience delivering oral PrEP—emphasised that pharmacy providers should have access to a clinician with PrEP expertise for on-demand consultations and referrals. Some reported that routine technical assistance and continuing medical education on LAI PrEP delivery would be key to safeguarding quality of care and recommended that training updates carry continuing professional development points, which pharmacy providers need to accrue annually for re-licensing.

#### Compatibility

Participants identified four grey areas that could influence how well LAI PrEP delivery fits with how pharmacies typically operate.

##### Procurement

Providers and stakeholders noted the need to establish how pharmacies would procure LAI PrEP and at what price, anticipating that high cost and an overly burdensome procurement process could hinder pharmacies’ uptake. Contrary to how pharmacies in Kenya usually source their drugs—from a wide variety of distributors and manufacturers—some stakeholders recommended pharmacies be required to source LAI PrEP from the government or a limited set of authorised distributors to control drug quality and reduce the risk of stock-outs. Referencing the procurement arrangement used in prior pilot studies^33 34^—whereby pharmacies retrieved free oral PrEP from nearby public health facilities—several providers welcomed the idea of getting free LAI PrEP from government stock, but called for it to be delivered directly to their pharmacy, in line with how other products reach them.

##### Documentation and reporting

Stakeholders emphasised the importance of pharmacies documenting and reporting LAI PrEP services rendered both for monitoring and evaluation purposes and supply chain forecasting and quantification; however, several pointed out that maintaining detailed client records and completing routine MOH reports would be new for most pharmacies. Noting that pharmacies are not currently integrated into Kenya’s health information system, stakeholders highlighted the need to establish what metrics pharmacies would report on and how. A few anticipated that the time-consuming nature of these tasks would not fit well within pharmacy workflows, with one stakeholder recommending that existing reporting tools be pared down for pharmacies. Anticipating data quality issues, some stakeholders called for routine auditing of pharmacies’ LAI PrEP data and, where needed, supportive supervision. For pharmacovigilance reporting, providers and stakeholders felt that the existing electronic reporting system managed by the country’s drug regulatory authority would suffice.

##### Sharps disposal

Several providers felt that their pharmacy’s current way of disposing used sharps—bringing them to nearby public health facilities—would work well for LAI PrEP delivery; however, a few anticipated challenges—such as facilities not accepting the waste and the time burden associated with this task—and called for the MOH to ensure the existing sharps disposal system is reliable and to add a pick-up service.

##### Client follow-up

When asked about appointment reminders for LAI PrEP clients, most providers felt their current practices—most commonly, noting the client’s next appointment date in their internal records and calling or texting those who miss their appointment—would suffice; however, some providers and stakeholders noted that this follow-up approach relies on having up-to-date contact information for the client, yet clients often change phone numbers without notifying the pharmacy. Provider and stakeholder recommendations to mitigate this barrier included issuing appointment cards and asking clients to provide additional contact information, such as their home address, email or the phone number of a trusted friend or next of kin. One provider recommended pharmacies be equipped with an automated SMS appointment reminder system. One stakeholder similarly called for automated appointment reminders and additionally recommended collaboration with community health workers:

Human beings are prone to forgetting. So we don’t want somebody writing on a book that, ‘I need to remember to call this person after six months’. There’s a high likelihood that they will not remember. So how do we leverage on digital systems to support with [appointment] reminders? … My recommendation would be for the pharmacy to invest in good follow-up systems. … [It could involve] phone calls and figuring out how to link to existing community systems. Like the community health promoters—if they can get the unit [address] where the person is staying, … [they could] do [at-home] follow-up just to find out whether the client is still at risk or are they covered using any other HIV prevention methods. (*Female stakeholder from implementing organisation, age 38 years, ID #46*)

### Available resources

When discussing the supplies and equipment they anticipated needing to deliver LAI PrEP (table 4), providers reported that most items would be easy to acquire and/or keep in stock, but several noted that pharmacies’ willingness and ability to invest in these items would depend on the demand for and profitability of LAI PrEP. A few providers and stakeholders recommended the government support pharmacies with the requisite commodities (including the LAI PrEP drug) for free or at a subsidised cost to make delivery economically worthwhile for pharmacies and affordable for clients:

[The goal should be] to subsidize [the cost of] their [LAI PrEP] products to almost zero. That way, pharmacies do not have to pass all of the cost [on] to a client. They can … charge [clients] for the service [ie, the pharmacy provider’s time], but not for the products. (*Female stakeholder from pharmacy professional society, age 37 years, ID #44*)

**Table 4:** Pharmacy supplies and pharmacy provider competencies needed for injectable PrEP delivery

Pharmacy supplies	Pharmacy provider competencies
HIV testing kits Injectable PrEP drug Syringes Alcohol swabs/sanitiser Gloves Sharps disposal box and bins for biohazardous waste Exam table or couch Logbook Airtime for following up with clients Informational brochures for clients Job aids (eg, provider handbook)	Injectable PrEP guidelines Proper drug storage Counselling on HIV prevention options, including multiple PrEP products HIV testing and counselling Injection administration (theory and practice) Biohazardous waste disposal Handling adverse reactions Side effects monitoring Pharmacovigilance PrEP specialist consultation and referrals

PrEP, pre-exposure prophylaxis.

### Individuals domain

#### Client need

Participants from all three groups felt that availing LAI PrEP at pharmacies would fulfil a critical need among clients for HIV services that are accessible, fast, private and respectful. Accessibility was largely viewed as a byproduct of pharmacies’ proximity to where people live and work and their long operating hours. Fast service times were attributed to pharmacies’ short queues and few, if any, hand-offs between providers. Privacy would be protected, in part, by the fact that pharmacies offer a broad array of products and services for a wide range of health needs (not specifically HIV) and by the LAI PrEP delivery occurring in a non-descript room, precluding other customers from knowing what service the client received. Lastly, a few clients and providers asserted that pharmacy providers generally treat clients with more patience and kindness than their overworked, clinic-based counterparts and might, therefore, satisfy some clients’ need for respectful and non-judgemental treatment.

#### Client capability

Participants from all three groups identified low client knowledge about LAI PrEP and its availability at pharmacies as potential barriers to service uptake, with participants recommending a variety of approaches to increase client awareness, including social media, TV and radio ads; print materials, like posters and brochures; and in-person talks at clinics and community events.

#### Client opportunity

Across all three groups, participants said that clients’ opportunity to engage in pharmacy-delivered LAI PrEP services would be influenced by the cost of services as well as the quantity and distribution of pharmacies offering this service. Concerned about what pharmacies would charge for LAI PrEP, clients and stakeholders underscored the need for it to be ‘affordable’. Most clients thought LAI PrEP should be free or cost 100 Kenyan shillings (~US$1) at most, anticipating that any higher cost would price out a large portion of the target population. A few stakeholders felt that, if the government supplied pharmacies with the LAI PrEP drug for free, it could impose caps on how much, if anything, pharmacies charge clients. Several clients and providers also noted that, if LAI PrEP were only available at a handful of pharmacies, clients who travel frequently for school or work would have difficulty returning for follow-up doses; they, therefore, proposed making LAI PrEP available at pharmacies across the country.

#### Client motivation

Participants from all groups, especially clients and providers, predicted that clients’ motivation to engage in pharmacy-delivered LAI PrEP services would be influenced by their trust in the drug and confidence in the provider’s integrity and competency. For the former, participants felt that, given LAI PrEP’s newness, it would be especially important to assure people that the drug is safe, effective and government-approved. For the latter, participants (in particular, clients) warned of ‘quacks’—unlicensed or underqualified individuals who provide poor quality services—and asserted that clients would only be willing to get LAI PrEP from a provider whom they trusted would maintain confidentiality and provide genuine (not counterfeit) drugs:

[At some pharmacies] they may lie that they have given you that medication, but maybe it’s not the [right] one. They should show us the medicine before so that I can see the [expiration] date … [and ensure] they don’t inject me if it’s expired. (*Female pharmacy PrEP client who did not refill, age 33 years, ID #15*)

Participants from all groups additionally thought that clients would consider whether the provider understands the injection and is capable of safe injection administration:

You have to find out if the person giving you the injection is well knowledgeable in that field, or are they just administering it for the sake [of making money]? … Someone who is qualified will not harm you. (*Female pharmacy PrEP client who refilled, age 25 years, ID #11*)

To help clients identify qualified LAI PrEP providers, some providers recommended that providers receive and display a certificate of training. Concerning the professional cadre, about half of clients thought they would be comfortable getting CAB-LA from a trained pharmacy provider, whereas the remainder anticipated they would prefer a nurse or doctor. About one-third of clients specified they would only get LEN from a doctor or nurse in a clinic setting because they perceived the risk of harm to be greater given the abdominal injection site. Lastly, clients and providers said that some clients—especially those who fear judgement of their sexual activity—would take into account the provider’s bedside manner:

I would consider the pharmacy provider I am going to [and] if it is someone who understands me. … It is important to speak to clients well and encourage them … [and not] start telling them, ‘What you are doing is not good. Even if you are using PrEP, you should not [have multiple sex partners]’. (*Female clinic PrEP client, age 28 years, ID #17*)

#### Provider capability

Most providers and stakeholders felt as though, with proper training and support, pharmacy providers would be capable of safely delivering this service. Table 4 summarises the knowledge, skills and competencies that participants from these groups thought pharmacy providers would need to deliver LAI PrEP. Providers anticipated that administering LEN would be easy, noting that it is not uncommon for pharmacy providers to give subcutaneous injections after receiving on-the-job training and that some have already been trained by the MOH to deliver injectable contraception. A handful of providers said the government could consider requiring pharmacies to have nurses and/or doctors on staff to administer the injection, but that acquiring such staff would be cost-prohibitive for most pharmacies.

#### Provider opportunity

Nearly all providers expressed concern that the amount of time it takes to deliver LAI PrEP would exceed what pharmacy providers typically have available. Most of those with prior experience delivering oral PrEP said it was time-consuming and especially challenging during peak business hours. These providers worried that counselling clients on multiple forms of PrEP—oral, CAB-LA and/or LEN—would be even more time-consuming, and a few anticipated they would push clients to pick the form that was fastest for them to deliver. Some felt LEN—with only two visits per year—would be more feasible for pharmacy providers to deliver. Providers suggested several ways to mitigate barriers to provider availability, including pharmacies having two providers per shift; delivering LAI PrEP by appointment and the MOH seconding staff to pharmacies.

#### Provider motivation

Providers and stakeholders anticipated that the primary motivator for pharmacy providers to deliver LAI PrEP would be the prospect of financial gain. Participants from both groups pointed out that having a provider attend to LAI PrEP clients in a back room could lead to missed sales—and lost profit—for the pharmacy. A few providers further noted that, in some pharmacies, the owner incentivises employees by paying bonuses to those who hit daily sales targets. These providers thought this compensation structure might disincentivise providers from delivering LAI PrEP, as many other products take less time to deliver and fetch higher profits:

Here [at this pharmacy], we work with [sales] targets. Everyone has their targets, so even if I am serving the client here [in the back room], I must make sales. If they [LAI PrEP clients] consume your time here, the more you are losing there. … The big challenge [with delivering oral PrEP] was the fact that it was time-consuming. (*Female pharm tech with prior PrEP delivery experience, age 38 years, ID #34*)

To mitigate this challenge, a few providers recommended the government or implementer pay a direct incentive to the provider delivering the service, rather than compensating pharmacy owners and leaving it up to them to decide how much, if anything, to share with the provider.

## Discussion

In this qualitative study, Kenyan oral PrEP users, pharmacy providers and other key stakeholders were generally supportive of the idea of pharmacy-delivered LAI PrEP and expressed cautious optimism that, with the right support and oversight, this delivery channel could increase LAI PrEP access. Participants identified several characteristics of pharmacies that might facilitate LAI PrEP implementation, such as long operating hours; proximity to where people live and work; discrete service delivery and lack of HIV stigma. Participants also identified barriers at multiple levels that could hinder the acceptability, feasibility and/or adoption of LAI PrEP among pharmacy clients and providers. Findings from this research can inform decision-making about whether and how to expand LAI PrEP services to private pharmacies, including selection of implementation strategies that might best position this delivery channel for success in Kenya and similar settings.

Although many of the anticipated barriers identified in this formative research echo those reported in other LAI PrEP studies and real-world programmes,^1848^,^52^ a subset of these barriers is likely to be more pronounced in private pharmacies than in traditional healthcare delivery channels.^22^ These potential ‘deal breakers’ for pharmacy delivery include gaps in enabling pharmacy policy; exclusion of private pharmacies from LAI PrEP supply chains and health information systems; disincentives for private pharmacy providers to dedicate time to LAI PrEP delivery and clients’ general wariness of the private pharmacy sector, which has historically been plagued by unlicensed outlets with unqualified staff and/or counterfeit drugs.^53 54^ In private pharmacies, providers face low provider-to-client ratios and pressure to make sales—a core mandate of any for-profit business. These circumstances could undermine the availability and quality of pharmacy-delivered LAI PrEP services, with busy providers turning clients away or cutting corners on time-consuming delivery components, like choice-based counselling.^55^ To mitigate this risk, implementation strategies that reduce the amount of time pharmacy providers spend on delivery, such as availing staff who can assist with delivery in person or via telehealth,^35 56^ should be considered. To build client trust in this delivery channel, other strategies will be necessary, such as amending public registries of licensed pharmacies and pharmacy providers^25^ to indicate whether the premise or person is authorised to deliver LAI PrEP, and directing clients to apps they can use at the point of delivery to verify the authenticity of LAI PrEP drugs.^57^

As a later adopter of LAI PrEP, Kenya can capitalise on information that has already been generated about delivery in other settings. For example, several other LAI PrEP research studies and real-world programmes^1848^,^52^ have reported implementation barriers similar or identical to those anticipated by our study participants, such as low client knowledge of LAI PrEP and concerns about its safety; client travel as a barrier to attending follow-up visits and gaps in provider knowledge. Kenya could potentially leapfrog these common barriers by taking into consideration the implementation strategies that have worked well in other settings and leveraging and adapting existing resources for the pharmacy context, such as information, communication and education materials^18^; provider training curricula^58^,^61^; job aids^62^ and documentation and reporting tools.^22 63^ Importantly, several major implementation barriers that did not come up in this formative research have emerged in other settings and warrant careful consideration, such as type of HIV testing to be used at CAB-LA initiation and during CAB-LA use to identify acute infections or long-acting early viral inhibition (LEVI) syndrome,^64^ and how to ensure that clients who return late for CAB-LA re-doses (or who discontinue CAB-LA) use oral PrEP during delays (or after discontinuing) to reduce the risk of acquiring drug-resistant HIV.^65^

Synthesising this study’s findings with our team’s deep knowledge of the Kenyan HIV service delivery landscape and experience implementing oral PrEP delivery at over 100 private pharmacies across Kenya,^33^,^3543 66^ we drafted two example pathways for implementing LAI PrEP services at private pharmacies in Kenya in the short and long term (table 5). Although for illustrative purposes only, these example pathways highlight key, actionable areas for the Kenya MOH, implementing partners and/or civil society organisations to focus on as they plan for the introduction and scale-up of LAI PrEP. For expedited implementation in the short term, we suggest developing LAI PrEP certification programmes for pharmacy providers; granting special permission to pharmacies that already have PrEP prescribers on staff and seconding PrEP prescribers to pharmacies. For sustained implementation in the long term, we suggest revising national policies and guidelines (eg, PrEP implementation guidelines, pharmacy providers’ scope of practice); integrating pharmacies into Kenya’s health information system and amending public registries so clients can identify pharmacies and providers authorised to deliver LAI PrEP. Of note, these pathways are not mutually exclusive and could be pursued concurrently, especially since policy changes could take years to achieve.

**Table 5:** Potential implications for policy and practice: two example pathways for sustained and expedited implementation of pharmacy-delivered LAI PrEP services in Kenya

Barrier to pharmacy delivery*	Example actions	
	Long-term pathway for sustained implementation at scale	Short-term pathway for expedited implementation
Policy		
Gaps in enabling policies hinder legal delivery of injectable PrEP at pharmacies	Kenya MOH: Develop national injectable PrEP guidelines specifying which cadres of healthcare professional can deliver injectable PrEP. Revise the essential medicines list to indicate that injectable PrEP can be delivered at pharmacies meeting specific criteria. Revise the scope of practice of pharmacists and/or pharmaceutical technologists to include requisite injectable PrEP competencies (eg, permission to administer subcutaneous and/or intramuscular injections; prescribing privileges with remote clinician supervision).	Kenya MOH: Grant special permission to pharmacies with PrEP prescribers† on staff to initiate and manage clients on injectable PrEP. Second PrEP prescribers† to select pharmacies (eg, in HIV hot spots). Create an injectable PrEP certificate programme for pharmacy providers that confers permission to initiate and manage clients on injectable PrEP under remote clinician supervision.
Procurement and financing		
Private pharmacies are not currently part of Kenya’s PrEP supply chain system or pooled procurement mechanisms	Kenya MOH: Integrate pharmacies into the national PrEP supply chain (ie, issue them codes for ordering injectable PrEP directly from government warehouses). Determine compensation to private pharmacies (and/or allow pharmacies to charge clients) for injectable PrEP services, ensuring that the prospective profit is commensurate with delivery costs incurred. (*If applicable*) Establish a mechanism to pay pharmacies for injectable PrEP services rendered.	Kenya MOH: Link pharmacies to nearby public health facilities to obtain PrEP drugs and ancillary supplies (eg, HIV testing kits, needles, sharps disposal containers) from the national stock. Compensate pharmacies and limit what they can charge clients for injectable PrEP services.
Health Information System (HIS)		
Lack of interoperability between Kenya’s national HIS and those used by private pharmacies	Kenya MOH: Establish minimum criteria for HIS for injectable PrEP delivery. Identify specific HIS that could be used for injectable PrEP delivery, including for delivery at pharmacies.	Kenya MOH Implement the Kenya EMR PrEP module, currently used at select public health facilities offering CAB-LA, at private pharmacies.
Pharmacy provider availability and motivation		
Private pharmacies’ profit-generation focus and low staffing levels	Kenya MOH and/or implementing partners: Explore alternative financial arrangements (eg, pay-for-performance) that might motivate delivery of high-quality injectable PrEP services. Create legal pathways, as needed, for strategies found to reduce burden on pharmacy provider time (eg, telehealth scripting; HIV self-testing). Adapt/simplify injectable PrEP reporting tools for the pharmacy setting.	Kenya MOH and/or implementing partners: Second staff to pharmacies to assist with delivery in person or remotely. Consider direct financial incentives to individual pharmacy providers delivering services. Test implementation strategies that could reduce burden on pharmacy provider time (eg, option to self-screen for HIV risk; HIV self-testing with optional provider assistance; client-facing decision support tools).
Public confidence in the private pharmacy sector		
Client concerns about pharmacy providers’ competency and/or drug authenticity; public mistrust of the private pharmacy sector	Kenya MOH and implementing partners: Amend existing public registries of pharmacies and pharmacy providers to indicate which are authorised to deliver injectable PrEP. Create formal partnerships between private pharmacies and public health facilities.	Kenya MOH, implementing partners and civil society organisations: Create a temporary registry of pharmacy providers who have completed injectable PrEP training and issue them training certificates (to display in pharmacy) that clients can verify (eg, via QR code). Include information in demand generation materials about which pharmacies and providers are authorised to offer injectable PrEP and steps clients can take to verify this information at the point of delivery.

* These barriers are informed by, but not limited to, those reported by study participants and detailed in tables 2 and 3.

† Clinical officers or nurses working under remote clinician supervision.

CAB-LA, long-acting cabotegravir; EMR, electronic medical record; HIS, health information system; LAI, long-acting injectable; MOH, Ministry of Health; PrEP, pre-exposure prophylaxis.

Our study has limitations. First, because we limited our sample to pharmacy clients, pharmacy providers and select key stakeholders in two counties with high HIV prevalence, our findings do not likely reflect the full spectrum of factors that could help or hinder pharmacy-based LAI PrEP delivery in Kenya. Future research should solicit additional perspectives (eg, from key populations; donor agencies), including in geographies with different HIV epidemiological profiles. Second, we provided participants with standardised descriptions of CAB-LA and LEN to ensure their responses were anchored to a shared understanding of basic product facts; however, unmeasured factors—such as baseline knowledge about different types of injections—may have influenced participant responses, especially client concerns about LEN’s abdominal injection site. Future research should consider using visuals or demos on anatomical models. Third, our local research team’s positionality (in particular, their affiliation with KEMRI—the medical research arm of the Kenyan government) may have contributed to social desirability bias, such as pharmacy providers downplaying negative perspectives for fear of being excluded from future opportunities to deliver LAI PrEP. Lastly, because pharmacy-delivered LAI PrEP is hypothetical, participant perspectives may not accurately reflect how they would feel about the innovation in a real world scenario. Pilot studies are needed to evaluate pharmacy LAI PrEP delivery, including the experiences of clients and providers who experience the intervention first hand.

## Conclusion

Key stakeholders of HIV prevention in Kenya are cautiously optimistic that private pharmacies could be an effective delivery channel for LAI PrEP. Successful implementation will require integrating private pharmacies into multiple subsystems of Kenya’s public health sector and proactively addressing multilevel barriers. If private pharmacies facilitate access to and use of LAI PrEP, they could play a pivotal role in ending HIV as a public health threat.

## Supplementary material

10.1136/bmjgh-2025-019210 online supplemental file 1

10.1136/bmjgh-2025-019210 online supplemental file 2

## Data Availability

Data are available on reasonable request.
