# Supplementary material for: Implementing long-acting injectable HIV pre-exposure prophylaxis services at private pharmacies in Kenya: client, pharmacy provider and key stakeholder perspectives on potential challenges and opportunities
Source: BMJ Glob Health. 2026 Jan 28;11(1):e019210. doi: 10.1136/bmjgh-2025-019210 (PMC12853430; doi:10.1136/bmjgh-2025-019210)
Supplement: online supplemental file 1 [file bmjgh-11-1-s001.docx]

### BMJ Global Health Author Reflexivity Statement

Adapted from Morton, B., Vercueil, A., Masekela, R., Heinz, E., Reimer, L., Saleh, S., Kalinga, C., Seekles, M., Biccard, B., Chakaya, J., Abimbola, S., Obasi, A. and Oriyo, N. (2022), Consensus statement on measures to promote equitable authorship in the publication of research from international partnerships. Anaesthesia, 77: 264-276. <https://doi.org/10.1111/anae.15597>

| **Study conceptualisation** | |
| --- | --- |
| 1. How does this study address local research and policy priorities? | This study addresses local research and policy priorities by generating timely evidence on long-acting injectable (LAI) PrEP products. Specifically, this study explores the potential barriers and facilitators to offering LAI PrEP products at private pharmacies: a differentiated service delivery channel of high interest to the Kenya Ministry of Health (MOH), as per recent government publications, such as the Kenya Private Sector Engagement Framework for Delivery of HIV Services in Kenya 2023-2028 (published in March 2023).  At the time this research was undertaken (July to September 2023), Kenya’s drug regulatory authority was reviewing long-acting cabotegravir (CAB-LA) for HIV prevention, and, in anticipation of CAB-LA’s approval, the Kenya MOH was drafting national guidelines and a plan for CAB-LA’s roll-out. As described in our paper’s introduction section, Kenya approved CAB-LA in June 2024; however, supply shortages have delayed its national roll-out. Kenya has yet to approve lenacapavir (LEN), but in anticipation of its approval, the MOH has begun preparing for its introduction. As described in our paper’s discussion section, the MOH plans to take a total market approach, availing LEN both in public- and private-sector healthcare delivery venues. Our study findings may inform decision-making around whether and how to implement LAI PrEP products via this delivery channel in Kenya. |
| 1. How were local researchers involved in study design? | As noted in our paper’s Author Contribution Statement, the following researchers from the Kenya Medical Research Institute (KEMRI)—specifically, the Centre for Microbiology Research (CMR) and the Centre for Clinical Research (CCR/Partners in Health and Research Development)—were involved in study conception and design: Dr. Elizabeth A. Bukusi (co-PI), Dr. Kenneth Ngure, Dr. Victor Omollo, Dr. Felix Mogaka, and Kevin Kamolloh. These individuals also assisted with developing the study protocol and data collection instruments and obtaining necessary approvals to conduct this research in-country. |
| **Research management** | |
| 1. How has funding been used to support the local research team(s)? | Both KEMRI teams received funding in accordance with their submitted budgets, which were approved by the study funder (the Gates Foundation) and covered costs such as FTE for study personnel (e.g., co-investigators, research assistants, project coordinators) and benefits (e.g., health insurance); local and international travel; supplies (e.g., project office supplies, voice recorders); participant reimbursements; and overhead (e.g., utilities; internet). |
| **Data acquisition and analysis** | |
| 1. How are research staff who conducted data collection acknowledged? | The KEMRI researchers who conducted the in-depth interviews (authors NT, MO, VO, AO, PO, MH, and LA) are coauthors on this paper. Their role in data collection and analysis is also acknowledged in the Methods section of the paper. |
| 1. How have members of the research partnership been provided with access to study data? | Both KEMRI teams have full access to the study data. Per our IRB agreements, the KEMRI teams are responsible for the secure storage of identifiable data and only share de-identified versions of the interview transcripts with the team based at Fred Hutchinson Cancer Center (Seattle, USA). |
| 1. How were data used to develop analytical skills within the partnership? | The KEMRI researchers who conducted the in-depth interviews and participated in their analysis (authors NT, MO, VO, AO, PO, MH, and LA) had extensive prior experience with qualitative data analysis. (See Appendix C—COREQ checklist—for more details.) This study provided an opportunity to gain new skills—or hone existing skills—in rapid qualitative data analysis. |
| **Data interpretation** | |
| 1. How have research partners collaborated in interpreting study data? | The aforementioned research staff were deeply involved in data interpretation. The Methods section of our paper details how members of the analytic team noted their observations in the interview summaries and met routinely to discuss emerging patterns/themes and how best to package findings for dissemination, both in this manuscript as well as at a stakeholder meeting we hosted in January 2024. (For more details on this meeting, see item #9 below.) |
| **Drafting and revising for intellectual content** | |
| 1. How were research partners supported to develop writing skills? | Several KEMRI researchers were supported with writing abstracts for local and international conferences. For example, Alfred Odira presented a poster on lenacapavir findings from this study at HIVR4P 2024 in Lima, Peru, and Dr. Catherine Kiptinness presented a poster on this study’s findings at the 2025 annual meeting of the University of Nairobi’s Collaborative HIV/STI/Reproductive Health Research Group. A second manuscript from this dataset—on pharmacy delivery of the dapivirine vaginal ring—is currently under development by Valerie Ogello. |
| 1. How will research products be shared to address local needs? | In January 2024, we disseminated our findings at a stakeholder meeting, attended by the following Kenyan organizations:   - Policymakers and regulators National AIDS & STI Control Program (NASCOP); Pharmacy and Poisons Board (PPB) - Pharmacy providers and professional bodies: Kenya Pharmaceutical Association (KPA); Pharmaceutical Society of Kenya (PSK); Kenya Medical Association (KMA); Kenya Medical Laboratory Technology & Technicians Board (KMLTTB); Jomo Kenyatta University’s School of Pharmacy - Implementing partners: Jhpiego; Liverpool VCT (LVCT) Health; Center for International Health, Education, and Biosecurity (CIHEB) - Pharmacy clients and client advocacy groups: pharmacy clients; AIDS Vaccine Coalition (AVAC); WACI Health; ISHTAR; the Key Populations Consortium.   In addition to presentations on our study findings, the stakeholder meeting featured small and large group discussions to build consensus around next steps towards implementing pharmacy-based LAI PrEP delivery in Kenya.  Once published, we will share this paper with relevant LAI PrEP stakeholders, such as County and sub-County Health Management Teams (CHMTs) responsible for HIV prevention programming at the county and sub-county levels. |
| **Authorship** | |
| 1. How is the leadership, contribution and ownership of this work by LMIC researchers recognised within the authorship? | Of the 18 authors on this paper, 13 are Kenyan researchers. Their leadership, contribution, and ownership are further recognized in the paper itself, such as the sections on data collection and analysis and the Author Contributions Statement. |
| 1. How have early career researchers across the partnership been included within the authorship team? | The authorship team includes four researchers who had early stage investigator (ESI) status at the time of this study (Drs. Katrina Ortblad, Victor Omollo, Felix Mogaka, and Stephanie Roche); one researcher who was a PhD student at the time (Kevin Kamolloh); and eight researchers who have completed undergraduate or master’s degrees, several of whom plan to pursue doctoral-level research training: Nicholas Thuo, Maurice Opiyo, Vallery Ogello, Alfred Odira, Emmah Owidi, Perez Ochwal, Marion Hewa, and Lydia Adiema. |
| 1. How has gender balance been addressed within the authorship? | Just over half (11/18, 61%) of the authors identify as female. |
| **Training** | |
| 1. How has the project contributed to training of LMIC researchers? | KEMRI’s Centres for Microbiology (CMR) and Clinical Research (CCR) have led world-class HIV biomedical and implementation research since the 1980s, generating evidence that has shaped HIV prevention, treatment, and service delivery in Kenya and globally. KEMRI’s research partnerships emphasize mutual benefit and bidirectional learning. As noted in items #6 and 8 above, this project offered KEMRI researchers additional training in rapid qualitative data analysis, academic writing, scientific communication, and project administration, while collaborators from high-income countries benefited from KEMRI colleagues’ deep content expertise, community relationships, and professional networks, without which this research would not be possible. |
| **Infrastructure** | |
| 1. How has the project contributed to improvements in local infrastructure? | As mentioned in item #3 above, the KEMRI teams received funding to support their physical facilities and equipment (e.g., rent; utilities; building maintenance; tablets; voice recorders; office supplies) as well as their data/IT systems (e.g., internet; computers; project archival costs). |
| **Governance** | |
| 1. What safeguarding procedures were used to protect local study participants and researchers? | To protect local study participants and researchers, this study was overseen by two local Institutional Reviews Boards (IRBs): the Kenya Scientific Ethics Review Unit (SERU) subcommittees for CMR and CCR. It was also overseen by the University of Washington’s IRB. The study approval numbers are included in the paper. |
